# Supplementary material for: Patterns of Intron Gain and Loss in Fungi
Source: PLoS Biol. 2004 Nov 30;2(12):e422. doi: 10.1371/journal.pbio.0020422 (PMC532390; doi:10.1371/journal.pbio.0020422)
Supplement: Table S1 — Also available at http://genes.mit.edu/NielsenEtAl/. (4.3 MB ZIP). [file pbio.0020422.st001.zip › NielsenEtAl/html/963.html]

AN0824.1.NCU06543.1.MG08690.1.FG09661.1


```
 CLUSTAL W (1.82) Multiple Sequence Alignments - Introns Inserted


Sequence 1: NCU06543.1	433 aa
Sequence 2: FG09661.1	412 aa
Sequence 3: MG08690.1	400 aa
Sequence 4: AN0824.1	437 aa
Alignment Length: 437 aa
Number Identitical Residues: 283 aa
Alignment Score (without introns) 12087


MG08690.1 	-----~---------------------MQR2SP--~--~-------ARRFDLHDLTPTPI
NCU06543.1	MKSFT~RVLRPAVRRA----QAVSAPRLSR~TPVV~QC2ARTLSTTSPRRDITDLPPTPI
FG09661.1 	-MSLT2NTAR----------------LLST~T---~QC~RR-----NEHIDISEIPPTPI
AN0824.1  	MASIT~RVLRPLSRTVPSLRPAAIRPLASR~PLQS2TY~AFSTTCRRREADLTGLTPTPI
          	  : :     .     .:   :   .  .  .         : :    . *:  :.****

MG08690.1 	THLSETEAAMQEAVSKFANDVVLPKVRDMDEAETMDPSLVEQLFEQGLMGVEIPEEYGGA
NCU06543.1	THFSEVETAMAETVQKFANDVILPKVRDMDEAEAMDPAIVEQLFEQGIMGVEIPEEYGGA
FG09661.1 	THLSELETAMVDTVSKFATDVILPRARDMDEAEEMDPAVVEQLFEQGLMGIEIPEEYGGA
AN0824.1  	THLSETESMMAETVSKFAEEQIGPKVREMDEAETMDPALVEHLFEQGIMGIEIPEEFGGA
          	**:** *: * ::*.*** : : *:.*:***** ***::**:*****:**:*****:***

MG08690.1 	GMNFTSAIVGIEELARVDPSVSVLVDVHNTLVNTAIIKWGSAELKKRFLPRLATETVGSF
NCU06543.1	GMNFTAAIVCIEELARIDPSVSVMVDVHNTLVNTAVIRWGSEALKKKYLPKLATNTVGSF
FG09661.1 	GMNFTAAIIGIEELARADPSVSVLVDVHNTLCNTAIIKHGSTAIKKKWLPRLATNTVASF
AN0824.1  	GMNFTAAIVAIEELARVDPSVSVLVDVHNTLVNTAFIKWGDAQVQRTWLPKLATGTVGSF
          	*****:**: ****** ******:******* ***.*: *.  ::: :**:*** **.**

MG08690.1 	CLSEPVSGSDAFALATKATETSDGFKISGSKMWITNSMEADFFIVFANLNPDKGYKGITA
NCU06543.1	CLSEPVSGSDAFALATRAVETENGYKINGSKMWITNSMEADFFIVFANVDPSKGYRGITA
FG09661.1 	CLSEPVSGSDAFAMATRATETADGFKINGSKMWITNSKEADLFIVFANLDPSKGYKGISA
AN0824.1  	CLSEPVSGSDAFALQTKAEKTADGYKLNGSKMWITNSMEAGVFIVFANLNPSKGYKGITA
          	*************: *:* :* :*:*:.********* **..******::*.***:**:*

MG08690.1 	FIVEKGTKGFSIAKKEKKLGIRASSTCVINFDDVEIPKENLLGERGAGYKYAIGLLNEGR
NCU06543.1	FIVEKGMKGFSIAKKEKKLGIKASSTCVLNFDDVEVPKENILGKVGEGYKYAIGILNEGR
FG09661.1 	FLVEKGMKGFSIAKKEKKLGIRASSTCVINFDDVEIPKENLLGERGQGYKYAISILNEGR
AN0824.1  	FIVEKDTPGFSIAKKEKKLGIKASSTCVLNFDDVVIPKGNLLGEEGQGYKYAIGLLNEGR
          	*:***.  *************:******:***** :** *:**: * ******.:*****

MG08690.1 	IGIAAQMTGLALGAWENAAR2YCYNDRKQFGQFIGEFQGMQHQMAQSYVEIAAARALVYN
NCU06543.1	IGIGAQMTGLALGAWENAVK~YVWNDRKQFGQLVGEFQGMQHQIAQSYTEIAAARALVYN
FG09661.1 	IGIAAQMTGLALGAFDNAAR~YVWNDRKQFGSLVGEFQGMQHQLAQAYTEITAARALVYT
AN0824.1  	IGIAAQMTGLALGAWENAAK2YVWNDRRQFGQLVGEFQGMQHQIAQAYTEIAAARALVYN
          	***.**********::**.: * :***:***.::*********:**:*.**:*******.

MG08690.1 	AARKKEAGEDFVRDAAMAKLYASQVAGRVSGLAVEWMGGMGFVREGLAEKFFRDSKIGAI
NCU06543.1	AARKKEAGEDFVMDAAMAKLYASQVAGRVSGLAVEWMGGMGFVREGLAEKFFRDSKIGAI
FG09661.1 	AARKKEAGEDFVKDAAMAKLYASQVAGRVSGSAIEWMGGMGFVREGLAEKYFRDSKIGAI
AN0824.1  	AARKKEAGQDFVQDAAMAKLYASQVAGRVAGSAVEWMGGMGFVREGIAEKMFRDSKIGAI
          	********:*** ****************:* *:************:*** *********

MG08690.1 	YEGTSNIQ~LNTIAKSLQKEYTS
NCU06543.1	YEGTSNIQ~LNTIAKTLQKQYTA
FG09661.1 	YEGTSNIQ2LNTIAKLLQKEYTS
AN0824.1  	YEGTSNIQ~LQTIAKLLQKQYTR
          	******** *:**** ***:**
```
